# Differences in cortical structure between cognitively normal East Asian and Caucasian older adults: a surface-based morphometry study

**DOI:** 10.1038/s41598-020-77848-8

**Published:** 2020-12-01

**Authors:** Dong Woo Kang, Sheng-Min Wang, Hae-Ran Na, Sonya Youngju Park, Nak Young Kim, Chang Uk Lee, Donghyeon Kim, Seong-Jin Son, Hyun Kook Lim

**Affiliations:** 1 Department of Psychiatry, Seoul St. Mary’s Hospital, College of Medicine, The Catholic University of Korea, Seoul, Republic of Korea; 2 Department of Psychiatry, Yeouido St. Mary’s Hospital, College of Medicine, The Catholic University of Korea, Seoul, Republic of Korea; 3 Department of Radiology, Seoul St. Mary’s Hospital, College of Medicine, The Catholic University of Korea, Seoul, Republic of Korea; 4 Neurophet Inc., Seoul, Republic of Korea

**Keywords:** Cognitive ageing, Brain, Magnetic resonance imaging

## Abstract

There is a growing literature on the impact of ethnicity on brain structure and function. Despite the regional heterogeneity in age-related changes and non-uniformity across brain morphometry measurements in the aging process, paucity of studies investigated the difference in cortical anatomy between the East Asian and Caucasian older adults. The present study aimed to compare cortical anatomy measurements, including cortical thickness, volume and surface area, between cognitively normal East Asian (n = 171) and Caucasian (n = 178) older adults, using surface-based morphometry and vertex-wise group analysis of high-dimensional structural magnetic resonance imaging (MRI) data. The East Asian group showed greater cortical thickness and larger cortical volume in the right superior temporal gyrus, postcentral gyrus, bilateral inferior temporal gyrus, and inferior parietal cortex. The Caucasian group showed thicker and larger cortex in the left transverse temporal cortex, lingual gyrus, right lateral occipital cortex, and precentral gyrus. Additionally, the difference in surface area was discordant with that in cortical thickness. Differences in brain structure between the East Asian and Caucasian might reflect differences in language and information processing, but further studies using standardized methods for assessing racial characteristics are needed. The research results represent a further step towards developing a comprehensive understanding of differences in brain structure between ethnicities of older adults, and this would enrich clinical research on aging and neurodegenerative diseases.

## Introduction

Differences in ethnicity encompass genetic, lingual, cultural, and environmental factors, which all may translate to differences in brain structure and function. In particular, there is a growing literature comparing the functional activation of brain associated with language, social cognition, and stimuli processing between East Asians and Caucasians^1–4^.

Although heavily outnumbered by research comparing differences in brain function between Asians and Caucasians, few studies illustrated the effect of ethnicity on brain structure using high-resolution magnetic resonance imaging (MRI). Studies consistently reported that East Asian have greater cortical anatomy measurements in the frontal lobe, but controversial findings were reported regarding the effect of ethnicity on the cortical measurements of the temporal and parietal regions^4–6^. Previous studies further suggested that this difference may be attributed to the distinct language, environment, and culture of each ethnicity. However, sample sizes for all previous researches were small and their demographic factors were not matched properly which limited generalizability. Above all, it is difficult to apply the previous results to the older population because most of the studies were conducted in young adults.

Structural changes of the brain during aging process lead to a difference in the cortical anatomy between younger and older adults^7^. There is also regional heterogeneity and a specificity of measurements in age-related changes in brain morphometry^8–10^. Thus, accurate assessment of the differences in brain structure among older ethnic groups could deepen our understanding of neurodegenerative brain diseases that progress with age. Brain structural change may also be a valuable biomarker for early diagnosis of neurodegenerative diseases^11^, because changes in brain structure reflect pathological and cognitive changes over the trajectory of neurodegenerative disease, including dementia^12,13^. However, interpreting the results of brain structure changes without considering racial differences can lead to distorted understanding. Therefore, an accurate evaluation of the differences in brain structure among cognitively normal older ethnicities is needed to precisely analyze changes in brain structure related to neurodegenerative diseases according to different races^14^.

A software tool for predicting neurodegenerative diseases using brain structure was recently developed^15^. Differences in cortical anatomy between older ethnicities may affect reliability as a biomarker. Consideration is especially crucial for mapping the brain structure into a standard template to compare cortical anatomy between study groups, and there is increased recognition of the importance of age and ethnic-specific templates to improve the accuracy of brain imaging studies on neurodegenerative disease^16,17^.

Brain cortical anatomy is structured as a two-dimensional corrugated sheet of tissue, of which the surface-based model has been known to allow for better implementation^18^. This surface-based morphometry method has been demonstrated to be more sensitive to age-related brain structural changes than voxel based-morphometry^19^. Furthermore, surface-based morphometry can provide more specific morphometric features, including cortical thickness, cortical volume, and surface area^20,21^. The association between these features has been reported to be non-uniform^22^, while cortical thickness and surface area have been proposed to be genetically independent^23^. Therefore, comprehensive evaluation of cortical anatomy features may provide valuable information in the evaluation of structural changes of the brain.

The present study aimed to compare cortical anatomy measurements, including cortical thickness, volume, and surface area between cognitively normal East Asian and Caucasian older adults, using semi-automated brain morphometry tools and vertex-wise analysis. Compared to previous studies, a larger sample population was analyzed with matched age, sex, education years, and MMSE scores between ethnic groups.

## Materials and methods

### Participants

One hundred seventy-one East Asian subjects between 60 and 85 years old were included in this study. Subjects were recruited from the Catholic Brain Health Center MRI database, which was built through the outpatient psycho-geriatric clinic of Yeouido Saint Mary’s Hospital located in Seoul, Republic of Korea, from October 2018 through July 2020. The cognitive functions of all subjects were assessed with the Korean version of the Consortium to Establish a Registry for Alzheimer’s Disease (CERAD-K)^24^. Measures included assessment in verbal fluency (VF), the 15-item Boston Naming Test (BNT), the Korean version of the Mini-Mental State Examination (MMSE-K) (Park, 1989), Word List Memory (WLM), Word List Recall (WLR), Word List Recognition (WLRc), Constructional Praxis (CP), and Constructional Recall (CR). Inclusion criteria were as follows: (1) Participant with or without subjective memory complaints, beyond what would be expected for age, (2) Normal memory function documented by scoring above age, sex, and education adjusted cutoffs on the WLM, WLR, WLRc domain, (3) MMSE-K score between 24 and 30, (4) Clinical Dementia Rating = 0. Memory Box score must be 0, (5) Cognitively normal, based on the absence of significant impairment in cognitive functions or activities of daily living. All East Asian subjects were Korean, and only those who spoke Korean as their primary language were recruited. We excluded participants with any history of alcoholism, drug abuse, head trauma, or psychiatric disorders; those taking any psychotropic medications (e.g., cholinesterase inhibitors, antidepressants, benzodiazepines, and antipsychotics); those with multiple vascular risk factors; and those with extensive cerebrovascular disease. The study was conducted under the ethical and safety guidelines set forth by the Institutional Review Board of The Catholic University of Korea, which approved all research activity. Informed and written consent was obtained from all participants.

One hundred seventy-eight Caucasian subjects between 60 and 85 years old were included from the Alzheimer’s Disease Neuroimaging Initiative (ADNI)^25^. An individual was defined as cognitively normal when showing a MMSE score between 24 and 30 (inclusive), a CDR of 0, no signs of depression, and no objective memory loss (For further details on diagnostic guidelines and neuropsychological examinations please see the ADNI study website (https://adni.loni.usc.edu). In addition, only those who were classified as Caucasian in the racial category of the ADNI database and whose primary language was English were selected. The exclusion criteria were the same as the above.

### Data acquisition and preparation

Imaging data of the Catholic Brain Health Center MRI database were collected from the Department of Radiology of Yeouido Saint Mary’s Hospital at the Catholic University of Korea, using a 3 T Siemens Skyra MRI machine and a 32-channel Siemens head coil (Siemens Medical Solutions, Erlangen, Germany). One hundred seventy-one East Asian subjects were imaged with the T1-weighted magnetization-prepared rapid gradient-echo (MP-RAGE) sequence using the following parameters: image size = 224 × 224 × 256, voxel size = 0.9 × 0.9 × 0.9 mm^3^, repetition time (TR) = 1,940 ms, echo time (TE) = 2.6 ms, flip angle = 9°. MR images of the ADNI database were obtained using a Siemens (n = 266), Philips (n = 80), GE (n = 8) 3 T scanners. One hundred seventy-eight Caucasian subjects were imaged with the MP-RAGE sequence using the following parameters: image size = 192–256 × 192–256 × 160–211, voxel size = 1.00–1.25 × 1.00–1.25 × 1.00–1.20 mm^3^, repetition time (TR) = 1668–2300 ms, echo time (TE) = 2.52–3.25 ms, flip angle = 8°–10°. All brain images were globally co-registered to the ICBM152 brain template^26^, based on through a rigid-body transformation. A quality control (QC) procedure was performed by one radiologist on all raw T1-weighted images to check the motion artifacts or poor resolution of gray/white matter contrast.

### Morphometric analysis

#### Image processing

FreeSurfer software (version 6.0.0, https://surfer.nmr.mgh.harvard.edu) was used to reconstruct and co-register the cortical surfaces and estimate brain structural features including cortical thickness, cortical volume, and surface area. Image processing for the cortical model was done in the following order: removal of nonbrain tissue using a hybrid watershed algorithm^27^, bias field correction, automated Talairach transformation, segmentation of subcortical white matter and deep gray matter structures^28,29^, intensity normalization, tessellation of the gray/white matter boundary, and gray/cerebrospinal fluid (CSF) boundary, automated topology correction^27,30^, and surface deformation following intensity gradients to optimally place the gray/white and gray/CSF borders at the location where the greatest shift in intensity defines the transition to the other tissue class^20^. Individual cortical folding patterns were then registered to a spherical atlas in order to match cortical geometry across subjects. Thickness was calculated at each location of the cortex as the distance between the white matter and pial surface^20^. Procedures for the measurement of cortical thickness have been validated against histological analysis as well as manual measurements^31,32^. All data were smoothed with a 10 mm full width half maximum (FWHM) Gaussian kernel, and the cerebral cortex was parcellated based on gyral and sulcal information derived from manually traced brains^29,33^. The quality control process was performed as follows: 1. Correct the pial surfaces to remove any non-brain tissue, 2. Correct the white matter surfaces to include any missing white matter, 3. Correct the white matter surfaces to remove any errant grey matter. For this process, manual edits were performed using the FreeSurfer editing tools. These procedures are well prescribed in related papers^6,34^.

### Statistical analysis

Statistical analyses for demographic data were performed with R software (version 2.15.3). Assumptions of normality was tested for continuous variables using the Kolmogorov–Smirnov test. All of these showed normal distribution. Two sample t-test and chi-square (χ^2^) test were used to assess for differences between the East Asian and Caucasian groups in terms of demographic variables, mean cortical thickness, intracranial volume, and total surface area. All statistical analyses used a two-tailed level of 0.05 for defining statistical significance with a cluster-extent threshold of 100 mm^2^.

FreeSurfer software (Version 6.0.0, http://surfer.nmr.mgh.harvard.edu/) was used for group analysis. An identical processing pipeline was applied. Surface-based normalization was computed to map thickness, volume, and surface area data of each subject onto a common group space that allows comparison across subjects at homologous points on the cortex. Cortical anatomy measurements were then smoothed with FWHM = 10 mm, and the GLM was fitted to the data with age, gender, education, and MMSE score as covariates. In addition, to minimize the effect of different MR vendors on brain structural measurements, MR vendors were included as a covariate. Depending on the type of cortical anatomy measurement, either the mean cortical thickness, cortical volume, or surface area was also included as a covariate. Vertex-wise statistical test was performed to compare the differences of these cortical anatomy measurements between East Asian and Caucasian groups. Corrections for multiple comparisons were conducted to control the false discovery rate (FDR) at 0.05. The corrected significance map with *p* < 0.05 was then overlapped onto the *fsaverage* brain template surface for visualization.

## Results

### Demographic and clinical characteristics of the study participants

Table 1 shows the baseline demographic and clinical data for the East Asian and Caucasian groups. There were no significant differences in age, sex, or years of education. However, although the average MMSE score was 0.4 point, there was a significant difference between the two groups (*p* < 0.001). With regard to the cortical anatomy measurements, mean cortical thickness, and total cortical surface area did not differ significantl, but the Caucasian group showed larger intracranial volume than the East Asian group (*p* = 0.006).

**Table 1:** Demographic and clinical characteristics of Caucasians and East Asians.

	Caucasians (n = 178)	East Asians (n = 171)	*P* value
Age (years)	70.5 ± 4.2	69.7 ± 5.7	0.156
Sex (M:F, %)	39.9:60.1	46.8:53.2	0.233
Education (years)	15.6 ± 2.0	15.3 ± 2.0	0.086
MMSE	29.0 ± 0.7	28.6 ± 1.1	< 0.001
Mean cortical thickness (mm)	2.4 ± 0.1	2.4 ± 0.1	0.979
Intracranial volume (mm^3^)	3,017,724.3 ± 347,039.7	2,925,662.3 ± 274,082.0	0.006
Total cortical surface area (mm^2^)	163,522.4 ± 16,366.8	162,153.6 ± 14,738.6	0.413

Data are presented as mean ± SD unless indicated otherwise. MMSE, Mini Mental Status Examination.

### Differences in cortical thickness

**Figure 1:**
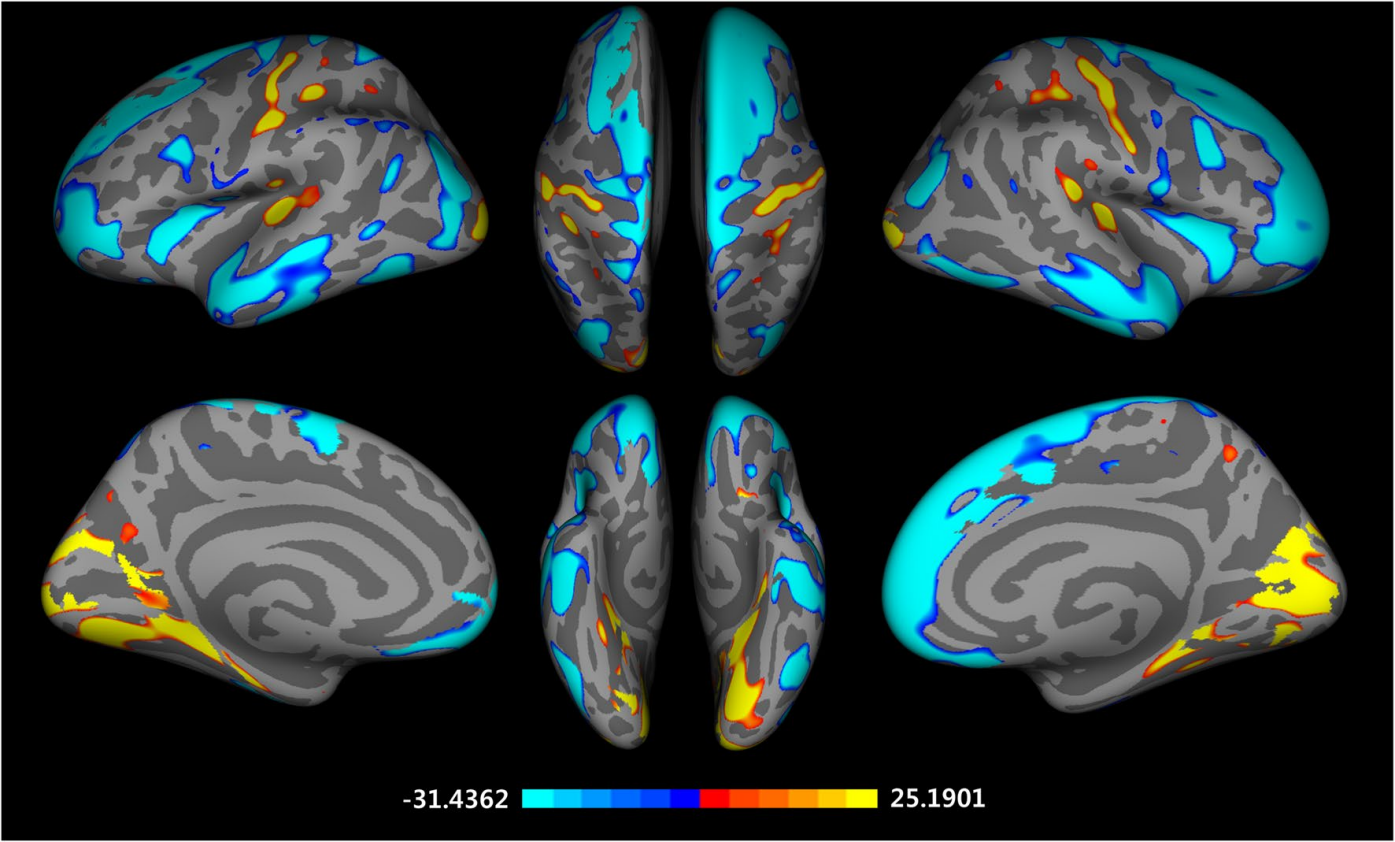
Vertex-wise group differences in cortical thickness adjusted for the effects of age, education years, MMSE scores, MR vendors, and mean cortical thickness (T-map, thresholded at FDR corrected *p* < 0.05, Caucasian-East Asian).

Significant vertex-wise differences between East Asian and Caucasian groups in cortical thickness are illustrated in Fig. 1. The East Asian group showed higher thickness in the bilateral superior frontal gyrus, caudal middle frontal gyrus, inferior parietal cortex, insula, inferior temporal gyrus, superior temporal gyrus, middle temporal gyrus, left paracentral lobule, precentral gyrus, fusiform gyrus, right postcentral gyrus, par orbitalis, medial orbital frontal cortex, and lingual gyrus (FDR corrected *p* < 0.05). The Caucasian group displayed higher thickness in the bilateral pericalcarine cortex, left transverse temporal cortex, lingual gyrus, cuneus cortex, postcentral gyrus, right lateral occipital cortex, and precentral gyrus than the East Asian group (FDR corrected *p* < 0.05). These findings are also found at 15 and 20 mm FWHM (Supplementary Fig. 1 online).

### Differences in cortical volume

**Figure 2:**
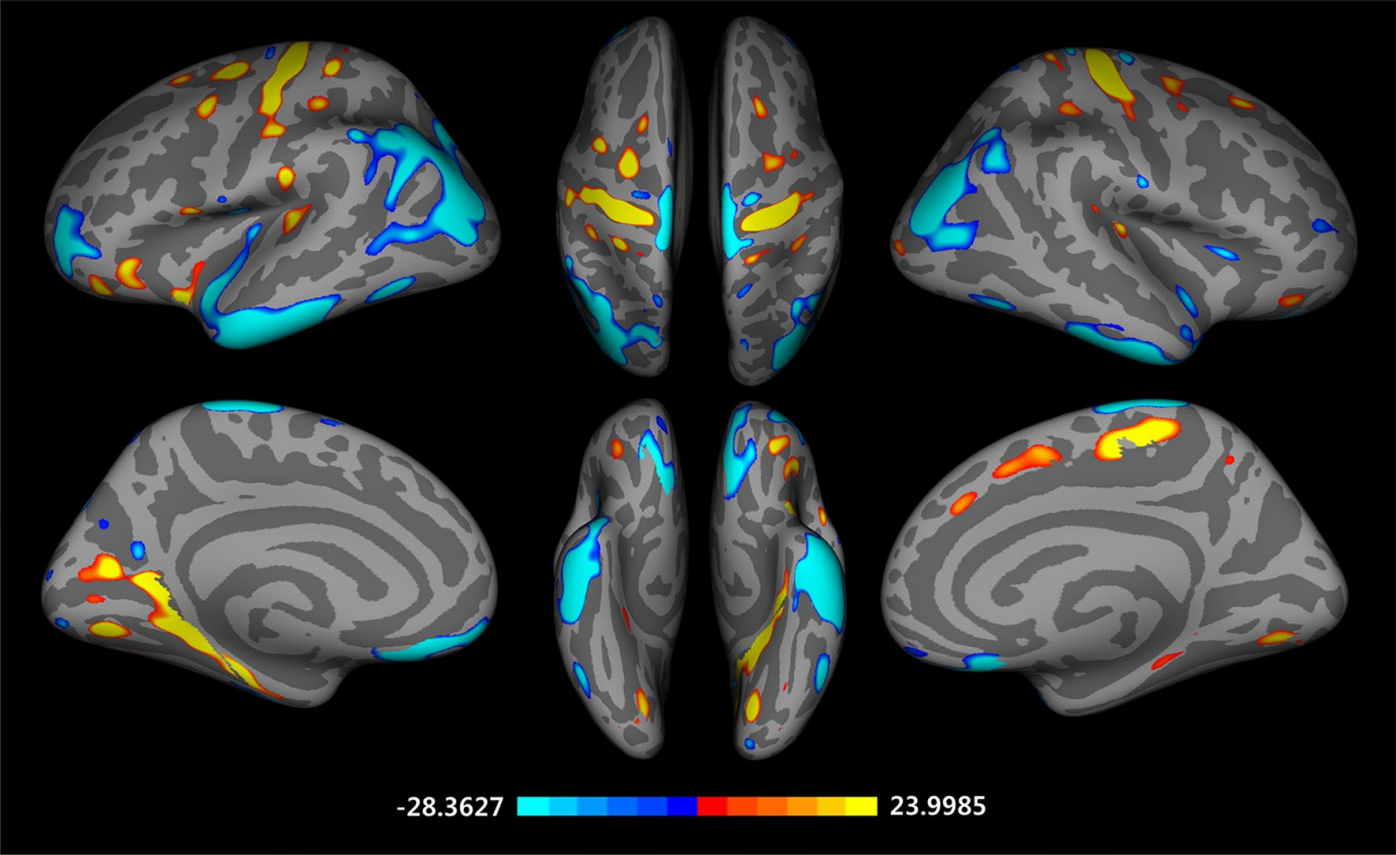
Vertex-wise group differences in cortical volume adjusted for the effects of age, education years, MMSE scores, MR vendors, and total intracranial volume (T-map, thresholded at FDR corrected *p* < 0.05, Caucasian-East Asian).

Figure 2 presents significant vertex-wise differences between East Asian and Caucasian groups in cortical volume. We found significantly larger cortical volume in the East Asian older adults in the bilateral inferior temporal gyrus, inferior parietal cortex, superior parietal cortex, postcentral gyrus, left rostral middle frontal gyrus, medial orbital frontal cortex, right temporal pole, superior temporal gyrus, and lateral orbital frontal cortex (FDR corrected *p* < 0.05). The Caucasian older adults showed a larger cortical volume in the bilateral precentral gyrus, left caudal middle frontal gyrus, isthmus–cingulate cortex, supramarginal gyrus, parahippocampal gyrus, pars opercularis, insula, transverse temporal cortex, lingual gyrus, right lateral occipital cortex (FDR corrected *p* < 0.05). These findings are also found at 15 and 20 mm FWHM (Supplementary Fig. 2 online).

### Differences in surface area

**Figure 3:**
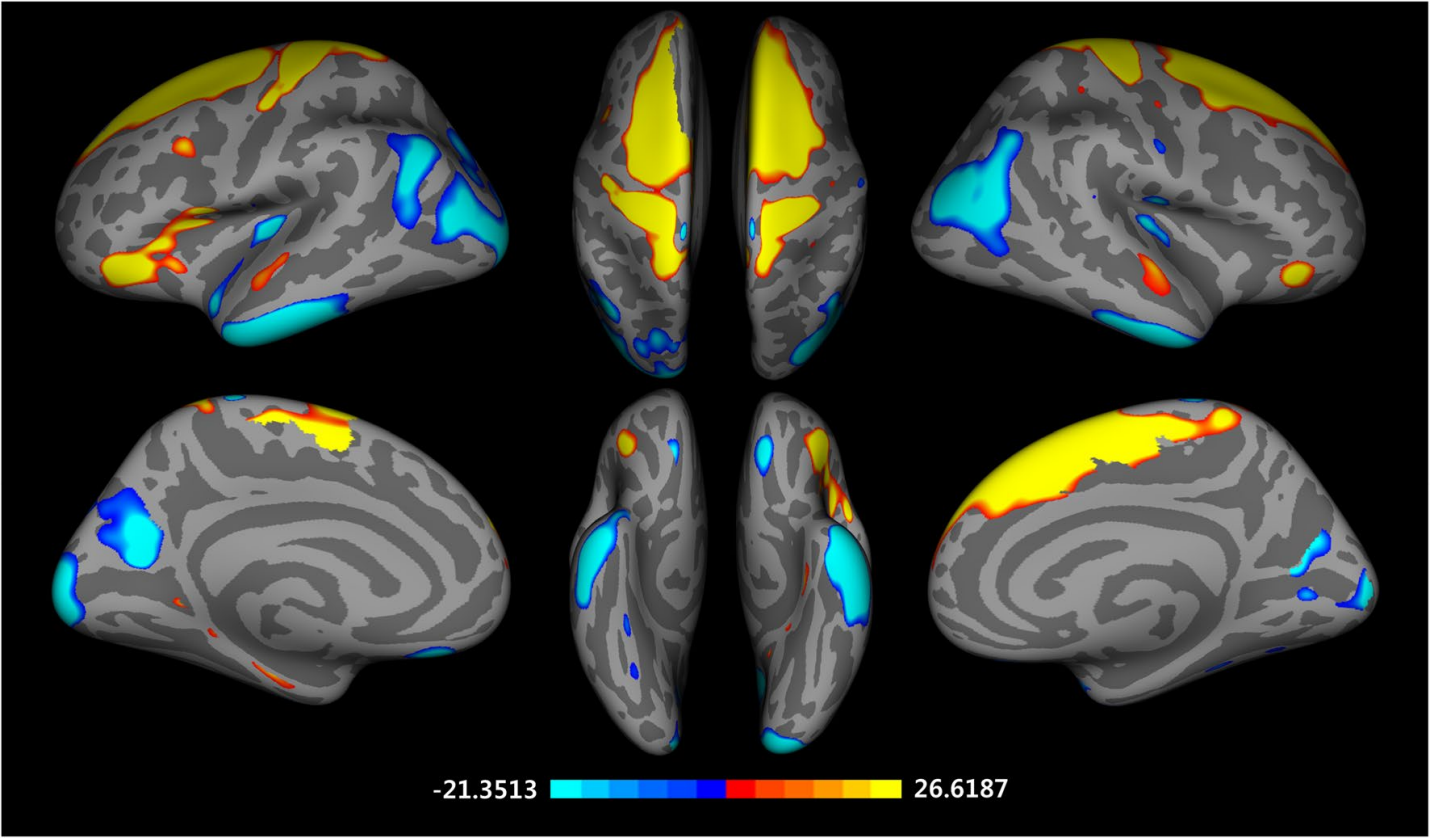
Vertex-wise group differences in cortical surface area adjusted for the effects of age, education years, MMSE scores, MR vendors, and total surface area (T-map, thresholded at FDR corrected *p* < 0.05, Caucasian-East Asian).

Significant vertex-wise differences between East Asian and Caucasian groups in cortical surface area are shown as Fig. 3. The East Asian group had a larger surface area in the bilateral inferior temporal gyrus, pericalcarine cortex, left superior parietal cortex, insula, precuneus, medial orbital frontal cortex, right postcentral gyrus, inferior parietal cortex, cuneus cortex (FDR corrected *p* < 0.05). The Caucasian group displayed larger surface area in the bilateral lateral occipital cortex, caudal middle frontal gyrus, left superior frontal gyrus, superior temporal gyrus, pars orbitalis, pars opercularis, parahippocampal gyrus, and right postcentral gyrus (FDR corrected *p* < 0.05). These findings are also found at 15 and 20 mm FWHM (Supplementary Fig. 3 online).

## Discussion

The present study was designed to examine differences in cortical anatomy measurements, including cortical thickness, volume, and surface area by surface-based morphometry and vertex-wise statistical analysis between cognitively normal East Asian and Caucasian older adults. The East Asian group showed greater cortical thickness and larger cortical volume in the right superior temporal gyrus, postcentral gyrus, bilateral inferior temporal gyrus, and inferior parietal cortex. The Caucasian group showed thicker and larger cortex in the left transverse temporal cortex, lingual gyrus, right lateral occipital cortex, and precentral gyrus. However, cortical regions with discordant differences in thickness and volume were also observed. The discrepancy between these cortical indicators was observed in previous studies^5,6^, and cortical thickness has been suggested to be more sensitive to the aging process^22,35^.

In accordance with the results herein, a previous study with young adults demonstrated that East Asians showed greater cortical thickness or larger volume in the superior frontal gyrus, middle, inferior temporal gyrus, and fusiform gyrus. Among these cortical regions, the inferior temporal and fusiform gyrus have been documented to be involved in visual processing, which is suggested to be influenced by ethnic difference^36,37^. Specifically, East Asians process visual information dependent on the context in a holistic way, while westerners interpret visual stimuli independent of the context in an analytic way^38,39^. With regard to the superior frontal and middle temporal gyrus, differences can be explained in part by language differences between ethnicities. It has been documented that the middle temporal gyrus is involved in the processing of sounds with complex spectral and temporal properties, which is a characteristic of Chinese^40^. Korean is known to have a linguistic similarity with Chinese^41^, and frontal and temporal cortex activation has been correlated with the phonological and semantic processing of Korean^42^. Although not described in previous studies showing racial differences, the inferior parietal cortex was found to be thicker and larger cortex in East Asians. This is in accord with earlier observations, which showed increased cortical density in Chinese speakers compared with in English speakers^43^. Moreover, the inferior parietal cortex has been documented to be activated when a Chinese speaker reads Chinese characters^44^. However, despite the similarities between Korean and Chinese mentioned above, there are also differences between the two languages. Chinese is composed of pictorial elements, but Korean has a fully alphabetic writing system with distinct signs for vowels and consonants. The results of the current study therefore need to be interpreted with caution.

The results of the present study with older subjects correspond well with earlier studies on younger participants, which reported that Caucasians displayed thicker or larger cortex in the precentral and postcentral gyrus^6^. The precentral gyrus is the site of the primary motor cortex^45^, and the postcentral gyrus is the location of the primary somatosensory cortex^46^. These cortical regions have been demonstrated to be robust against the aging process^47^. Although not identified in previous studies, the lateral occipital cortex was also found to show greater thickness and larger volume in Caucasians. This region has been reported to be activated in object-focused visual processing in the Caucasian elderly^48^. While cortical thickness of several regions including the bilateral Broca’s area, cingulate gyri, and the right precuneus showed ethnicity-related differences in an earlier study with young adults^6^, they were not observed in the current study with older adults. This discrepancy may be attributed to changes in the cortical structure between ethnicities in the aging process^47^. In summary, cultural differences and aging-insensitive cortical regions may account for some results of the present research.

The findings of the current study do not support previous literature on cognitively normal older adults, which classified the older adults into low and high performing groups based on processing speed in different ethnicities. There was no significant difference in cortical thickness between East Asians and Caucasians in the low-performing elderly, whereas high-performed elderly showed significant differences in several cortical regions^5^. However, among these regions which showed a significant difference in cortical thickness, only the inferior temporal gyrus was observed in the present study. The mean age of the subjects presented here was about 6 years older than those in the previous study, and the proportion of females was higher in the present research. Therefore, it is possible that these results were confounded by age and sex, both of which have been reported to affect cortical thickness^49,50^. Caution should be applied to the comparative analysis of these results, because the sample size of the previous study was only 1/6th of the present study.

In terms of surface area, only the left superior frontal cortex, which showed a larger surface area in the Caucasian older adults, was similarly noted in younger participants^6^. This discrepancy could again be attributed to differences on age, sex and sample size, as described above. Additionally, the difference in surface area between East Asians and Caucasians was discordant with cortical thickness of the bilateral pericalcarine, cuneus, caudal middle frontal cortex, left superior frontal, and superior temporal gyrus. This may be due to the negative association between the cortical thickness and surface area^47^, which has been indicated in a previous study suggesting that the expansion of the surface area might be an efficient way for compensating for cortical atrophy^51^.

Since the majority of previous studies have been on younger adults, changes in cortical anatomy in the aging process should be considered for interpreting the present findings. The prefrontal and parietal cortices have been known to be vulnerable to cortical atrophy in that these cortical regions have a compensatory role in normal aging and that functional over-recruitment of these regions in the aging process is correlated with local atrophy^22,52^. Furthermore, consideration should be given to the fact that the changes in each indicator of cortical anatomy during aging are nonuniform^22^, and that the rates of change across different brain regions vary^47^. Previous studies with young adults did not show significant differences in cortical thickness of the superior frontal gyrus and inferior parietal cortex, whereas Caucasians displayed greater thickness in the older adults herein. This discrepancy could be attributed to the accelerating atrophy in these cortical regions of East Asians in the aging process. Therefore, an understanding of the differences in the structural change of East Asian and Caucasian brains in the aging process is necessary for accurate interpretation of the current results. However, there is a current paucity of studies investigating this difference between ethnicities, and further research is warranted.

A number of limitations must be considered. First, there was a difference in image acquisition parameters and MRI scanners between the two groups because participants in this study were not recruited from the same center. Given that this also has an impact on the cortical anatomy measurements^53,54^, it adds further caution regarding the generalizability of the present findings. One of the major concerns is scanning parameter difference between groups. FreeSurfer driven measurements we utilized in this study, however, presented significant scanner-effects only for three out of the 18 cortical areas^55^. These noted three regions, left/right fusiform and the right superior frontal gyrus, did not overlap with our significant observations. Although MR vendors were included as a covariate and the identical processing pipeline was applied, these may not be sufficient to fully capture the scanner or site differences presented in the dataset we analyzed. Therefore, it is necessary to conduct further research either using the data from the homogenous sources or utilizing a new method to overcome data inhomogeneity.

We further acknowledge that the voxel size difference between MRI scans could have caused bias on the cortical measurements we investigated. The difference in voxel size, however, was in a minimal range 0.1–0.35 mm between two groups (0.9 mm versus 1.0–1.25 mm), which is smaller than measurable group difference (1 mm). Secondly, this study has described that the structural difference in specific brain regions between ethnicities means the difference in functions supported by the corresponding brain regions. This interpretation was based on the previous findings which have demonstrated that the brain regions with neuroanatomical differences show robust functional differences between ethnicities^4,56^. However, the lack of a standardized outcome measure for functional differences, such as language and information processing, makes it difficult to interpret the present results with confidence. This is also a recurring problem in previous studies comparing differences in brain structure between races^4–6^. Therefore, in further study, it is necessary to apply the standardized method for assessing racial characteristics and to recruit subjects within the same protocol. Thirdly, although Korean is more similar to Chinese in the language, environment, culture, and genetic factors than Caucasians, differences between the two still exist. Therefore, caution should be taken when comparing these results to previous studies with Chinese participant. Fourthly, there were significant differences in MMSE scores between two ethnicities. However, the difference in the mean MMSE scores was 0.4 points, so it was within the range that could be classified into the same category. Fifthly, the East Asian group in the present study included only Koreans. Therefore, further research including Chinese and Japanese should be carried out to validate the results of the current paper. Finally, for approaching the most accurate results, we would have to establish a cohort that can control all of the environmental and cultural factors that differ between different races, and other confounding factors. The cohort must undergo a strict process including setting the evaluation tool, MRI scanner type, and acquisition parameter identical among all multi-centers. Therefore, further works will be needed to more correctly study the difference in brain structure between races by establishing a more controlled cohort. In addition, the results of this study could be the starting point to get closer to the axiom of brain structural differences among different older races.

This study assessed the difference in cortical anatomy measurements by surface-based morphometry and vertex-wise analysis between cognitively healthy East Asian and Caucasian older adults. A significant difference in cortical thickness and volume was found in brain regions known to reflect cultural and language differences between ethnicities. However, other cortical regions did not overlap with brain regions that showed significant differences between East Asians and Caucasians in previous studies with young adults. Cortical anatomy is an important biomarker that reflects aging and neurodegenerative processes, and it has garnered attention as a potential moderator between neuropathology and clinical outcome of neurodegenerative disease^57^. Therefore, the comprehensive understanding of differences in brain structure between older ethnicities would provide an accurate reference for biomarkers and a template for structural, functional and molecular imaging, and enrich clinical research on aging and neurodegenerative diseases in multi-racial older adults.

## Supplementary information

Supplementary Figures.

## Data Availability

The datasets generated or analyzed during the current study are not publicly available due to Patient Data Management Protocol of Yeouido St. Mary’s Hospital but are available from the corresponding author on reasonable request. Demographic information, neuroimaging data, APOE genotype, CSF measurements, neuropsychological test scores, and diagnostic information are publicly available from the ADNI data repository (http://adni.loni.usc.edu

## References

[1] Hedden T, Ketay S, Aron A, Markus HR, Gabrieli JD (2008). Cultural influences on neural substrates of attentional control. Psychol. Sci..

[2] Zhu Y, Zhang L, Fan J, Han S (2007). Neural basis of cultural influence on self-representation. Neuroimage.

[3] Han S, Ma Y (2014). Cultural differences in human brain activity: a quantitative meta-analysis. NeuroImage.

[4] Kochunov P (2003). Localized morphological brain differences between English-speaking Caucasians and Chinese-speaking Asians: new evidence of anatomical plasticity. NeuroReport.

[5] Chee MWL, Zheng H, Goh JOS, Park D, Sutton BP (2011). Brain structure in young and old East Asians and Westerners: comparisons of structural volume and cortical thickness. J. Cogn. Neurosci..

[6] Tang Y (2018). Brain structure differences between C hinese and C aucasian cohorts: a comprehensive morphometry study. Hum. Brain Mapp..

[7] Kovalev VA, Kruggel F, von Cramon DY (2003). Gender and age effects in structural brain asymmetry as measured by MRI texture analysis. NeuroImage.

[8] Hogstrom LJ, Westlye LT, Walhovd KB, Fjell AM (2013). The structure of the cerebral cortex across adult life: age-related patterns of surface area, thickness, and gyrification. Cereb. Cortex.

[9] Dickerson BC (2009). Differential effects of aging and Alzheimer's disease on medial temporal lobe cortical thickness and surface area. Neurobiol. Aging.

[10] Pfefferbaum A (2013). Variation in longitudinal trajectories of regional brain volumes of healthy men and women (ages 10 to 85 years) measured with atlas-based parcellation of MRI. Neuroimage.

[11] Jack CR (2013). Tracking pathophysiological processes in Alzheimer's disease: an updated hypothetical model of dynamic biomarkers. Lancet Neurol..

[12] Villemagne VL (2013). Amyloid β deposition, neurodegeneration, and cognitive decline in sporadic Alzheimer's disease: a prospective cohort study. Lancet Neurol..

[13] Nagano-Saito A (2005). Cerebral atrophy and its relation to cognitive impairment in Parkinson disease. Neurology.

[14] Brickman AM (2008). Brain morphology in older African Americans, Caribbean Hispanics, and whites from northern Manhattan. Arch. Neurol..

[15] Tanpitukpongse TP, Mazurowski MA, Ikhena J, Petrella JR (2017). Predictive utility of marketed volumetric software tools in subjects at risk for Alzheimer disease: do regions outside the hippocampus matter?. Am. J. Neuroradiol..

[16] Fillmore PT, Phillips-Meek MC, Richards JE (2015). Age-specific MRI brain and head templates for healthy adults from 20 through 89 years of age. Front. Aging Neurosci..

[17] Shi L (2017). Using large-scale statistical Chinese brain template (Chinese2020) in popular neuroimage analysis toolkits. Front. Hum. Neurosci..

[18] Van Essen DC, Drury HA, Joshi S, Miller MI (1998). Functional and structural mapping of human cerebral cortex: solutions are in the surfaces. Proc. Natl. Acad. Sci..

[19] Hutton C, Draganski B, Ashburner J, Weiskopf N (2009). A comparison between voxel-based cortical thickness and voxel-based morphometry in normal aging. Neuroimage.

[20] Fischl B, Dale AM (2000). Measuring the thickness of the human cerebral cortex from magnetic resonance images. Proc. Natl. Acad. Sci..

[21] Fischl B, Sereno MI, Dale AM (1999). Cortical surface-based analysis: II: inflation, flattening, and a surface-based coordinate system. Neuroimage.

[22] 22Lemaitre, H. *et al.* Normal age-related brain morphometric changes: nonuniformity across cortical thickness, surface area and gray matter volume? *Neurobiol. Aging* **33**, e611–617. e619 (2012). 10.1016/j.neurobiolaging.2010.07.013 PMC3026893 20739099

[23] Panizzon MS (2009). Distinct genetic influences on cortical surface area and cortical thickness. Cereb. Cortex.

[24] Lee JH (2002). Development of the Korean Version of the Consortium to Establish a Registry for Alzheimer's Disease Assessment Packet (CERAD-K) clinical and neuropsychological assessment batteries. J. Gerontol. Ser. B: Psychol. Sci. Soc. Sci..

[25] Petersen RC (2010). Alzheimer's disease neuroimaging initiative (ADNI): clinical characterization. Neurology.

[26] Fonov V (2011). Unbiased average age-appropriate atlases for pediatric studies. Neuroimage.

[27] Ségonne F (2004). A hybrid approach to the skull stripping problem in MRI. Neuroimage.

[28] Fischl B (2002). Whole brain segmentation: automated labeling of neuroanatomical structures in the human brain. Neuron.

[29] Fischl B (2004). Sequence-independent segmentation of magnetic resonance images. Neuroimage.

[30] Fischl B, Liu A, Dale AM (2001). Automated manifold surgery: constructing geometrically accurate and topologically correct models of the human cerebral cortex. IEEE Trans. Med. Imaging.

[31] Rosas H (2002). Regional and progressive thinning of the cortical ribbon in Huntington’s disease. Neurology.

[32] Kuperberg GR (2003). Regionally localized thinning of the cerebral cortex in schizophrenia. Arch. Gen. Psychiatry.

[33] Desikan RS (2006). An automated labeling system for subdividing the human cerebral cortex on MRI scans into gyral based regions of interest. Neuroimage.

[34] Fischl B (2012). FreeSurfer. Neuroimage.

[35] Fjell AM (2009). High consistency of regional cortical thinning in aging across multiple samples. Cereb. Cortex.

[36] Haxby JV, Hoffman EA, Gobbini MI (2000). The distributed human neural system for face perception. Trends Cogn. Sci..

[37] Gold JM (2014). The perception of a familiar face is no more than the sum of its parts. Psychon. Bull. Rev..

[38] Goh JO (2010). Culture differences in neural processing of faces and houses in the ventral visual cortex. Soc. Cogn. Affect. Neurosci..

[39] Masuda T, Gonzalez R, Kwan L, Nisbett RE (2008). Culture and aesthetic preference: comparing the attention to context of East Asians and Americans. Pers. Soc. Psychol. Bull..

[40] Zahn R (2000). Hemispheric lateralization at different levels of human auditory word processing: a functional magnetic resonance imaging study. Neurosci. Lett..

[41] 41Huang, J.-X. & Choi, K.-S. in *Proceedings of the 38th Annual Meeting of the Association for Computational Linguistics.* 392–399.

[42] Yoon HW, Cho K-D, Park HW (2005). Brain activation of reading Korean words and recognizing pictures by Korean native speakers: A functional magnetic resonance imaging study. Int. J. Neurosci..

[43] 43Green, D. W., Crinion, J. & Price, C. J. Exploring cross-linguistic vocabulary effects on brain structures using voxel-based morphometry. *Bilingualism: Lang. Cognition* **10**, 189–199 (2007). 10.1017/s1366728907002933 PMC2312335 18418473

[44] Tan LH, Laird AR, Li K, Fox PT (2005). Neuroanatomical correlates of phonological processing of Chinese characters and alphabetic words: A meta-analysis. Hum. Brain Mapp..

[45] Rao SM (1995). Somatotopic mapping of the human primary motor cortex with functional magnetic resonance imaging. Neurology.

[46] Geyer S, Schormann T, Mohlberg H, Zilles K (2000). Areas 3a, 3b, and 1 of human primary somatosensory cortex: 2. Spatial normalization to standard anatomical space. Neuroimage.

[47] Storsve AB (2014). Differential longitudinal changes in cortical thickness, surface area and volume across the adult life span: regions of accelerating and decelerating change. J. Neurosci..

[48] Goh JO (2007). Age and culture modulate object processing and object—scene binding in the ventral visual area. Cogn. Affect. Behav. Neurosci..

[49] Hurtz S (2014). Age effects on cortical thickness in cognitively normal elderly individuals. Dementia Geriatric Cogn. Disorders Extra.

[50] Sowell ER (2007). Sex differences in cortical thickness mapped in 176 healthy individuals between 7 and 87 years of age. Cereb. Cortex.

[51] Murre J, Sturdy DP (1995). The connectivity of the brain: multi-level quantitative analysis. Biol. Cybern..

[52] 52Kalpouzos, G., Persson, J. & Nyberg, L. Local brain atrophy accounts for functional activity differences in normal aging. *Neurobiol. Aging* **33**, e621–623. e613 (2012). 10.1016/j.neurobiolaging.2011.02.021 21524432

[53] Wonderlick J (2009). Reliability of MRI-derived cortical and subcortical morphometric measures: effects of pulse sequence, voxel geometry, and parallel imaging. Neuroimage.

[54] Han X (2006). Reliability of MRI-derived measurements of human cerebral cortical thickness: the effects of field strength, scanner upgrade and manufacturer. Neuroimage.

[55] Jovicich J (2013). Brain morphometry reproducibility in multi-center 3 T MRI studies: a comparison of cross-sectional and longitudinal segmentations. Neuroimage.

[56] Anurova I, Renier LA, De Volder AG, Carlson S, Rauschecker JP (2015). Relationship between cortical thickness and functional activation in the early blind. Cereb. Cortex.

[57] Bartrés-Faz D, Arenaza-Urquijo EM (2011). Structural and functional imaging correlates of cognitive and brain reserve hypotheses in healthy and pathological aging. Brain Topogr..

