# Supplementary material for: Differences in cortical structure between cognitively normal East Asian and Caucasian older adults: a surface-based morphometry study
Source: Sci Rep. 2020 Dec 1;10:20905. doi: 10.1038/s41598-020-77848-8 (PMC7708477; doi:10.1038/s41598-020-77848-8)
Supplement: Supplementary file 1 — Supplementary Figures. [file 41598_2020_77848_MOESM1_ESM.pdf]

Supplementary Material

Differences in cortical structure between cognitively normal East Asian and Caucasian older adults: A surface-based morphometry study

Dong Woo Kang[1], Sheng-Min Wang[2], Hae-Ran Na[2], Sonya Youngju Park[3]

Nak Young Kim[2], Chang Uk Lee[1], Donghyeon Kim[4], Seong-Jin Son[4], Hyun Kook Lim[2*]

[1]Department of Psychiatry, Seoul St. Mary's Hospital, College of Medicine, The Catholic University of Korea, Seoul, Republic of Korea

[2]Department of Psychiatry, Yeouido St. Mary's Hospital, College of Medicine, The Catholic University of Korea, Seoul, Republic of Korea

[3]Department of Radiology, Seoul St. Mary's Hospital, College of Medicine, The Catholic University of Korea, Seoul, Republic of Korea

[4]Neurophet Inc., Seoul, Republic of Korea

***Correspondence:** Hyun Kook Lim, MD, PhD

Department of Psychiatry, Yeouido St. Mary's Hospital, College of Medicine, The Catholic University of Korea, 10, 63-ro, Yeongdeungpo-gu, Seoul, 06591, Republic of Korea

Tel: +82-2-3779-1048, Fax: +82-2-780-6577, E-mail: drblues@catholic.ac.kr

Supplementary Figure Legends

Supplementary Figure S1 Vertex-wise group differences in cortical thickness adjusted for the effects of age, education years, MMSE scores, MR vendors, and mean cortical thickness (T-map, thresholded at FDR corrected $p < 0.05$, Caucasian-East Asian)

(A) Images smoothed at 15mm full width at half maximum

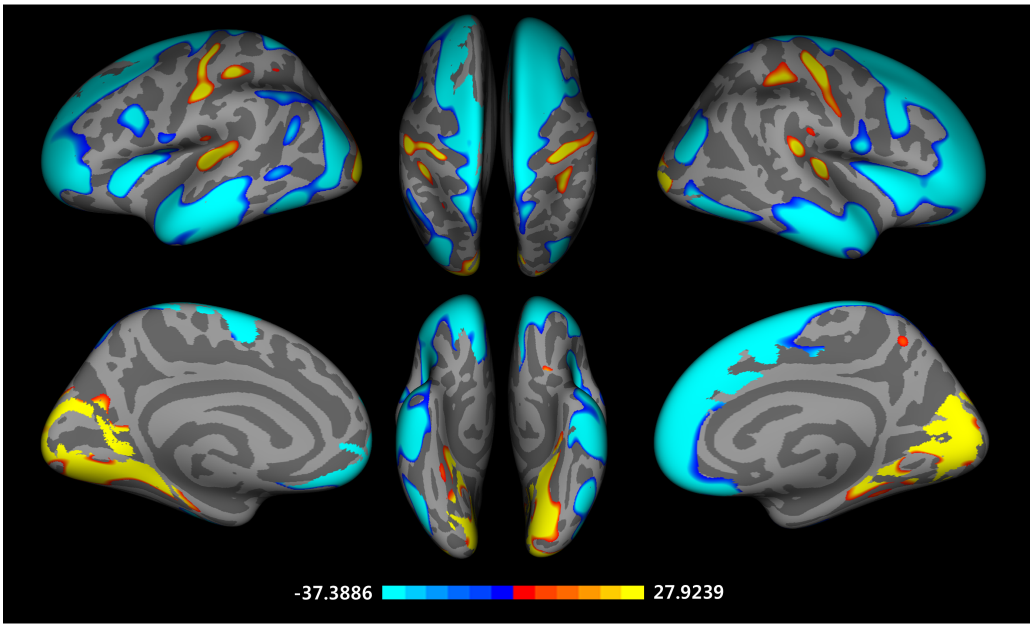

(B) Images smoothed at 20mm full width at half maximum

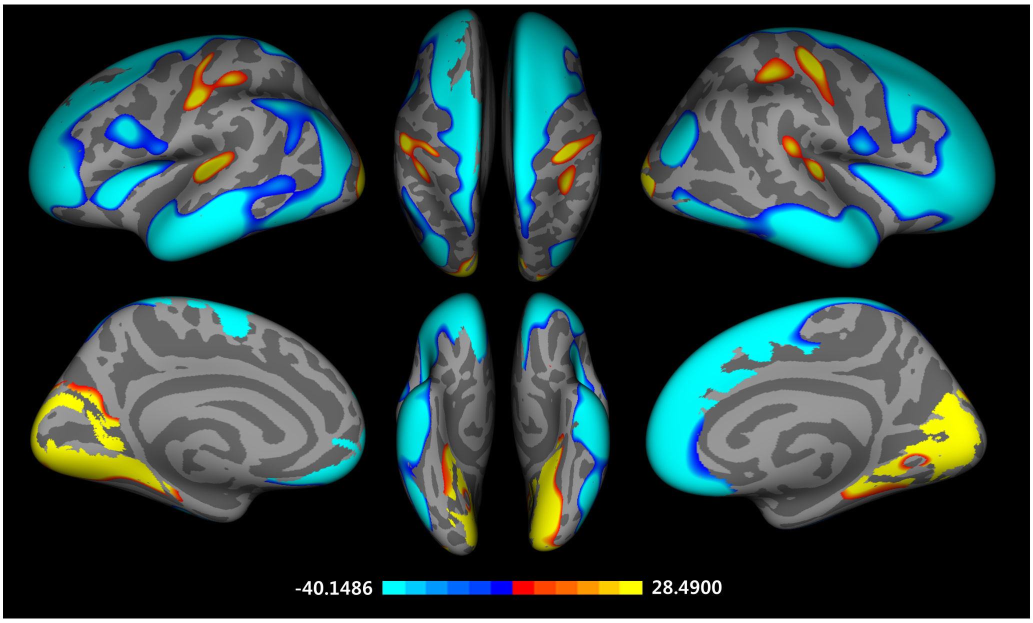

These images were created using FreeSurfer software (Version 6.0.0, http://surfer.nmr.mgh.harvard.edu/)

Supplementary Figure S2 Vertex-wise group differences in cortical volume adjusted for the effects of age, education years, MMSE scores, MR vendors, and total intracranial volume (T-map, thresholded at FDR corrected $p < 0.05$, Caucasian-East Asian)

(A) Images smoothed at 15mm full width at half maximum

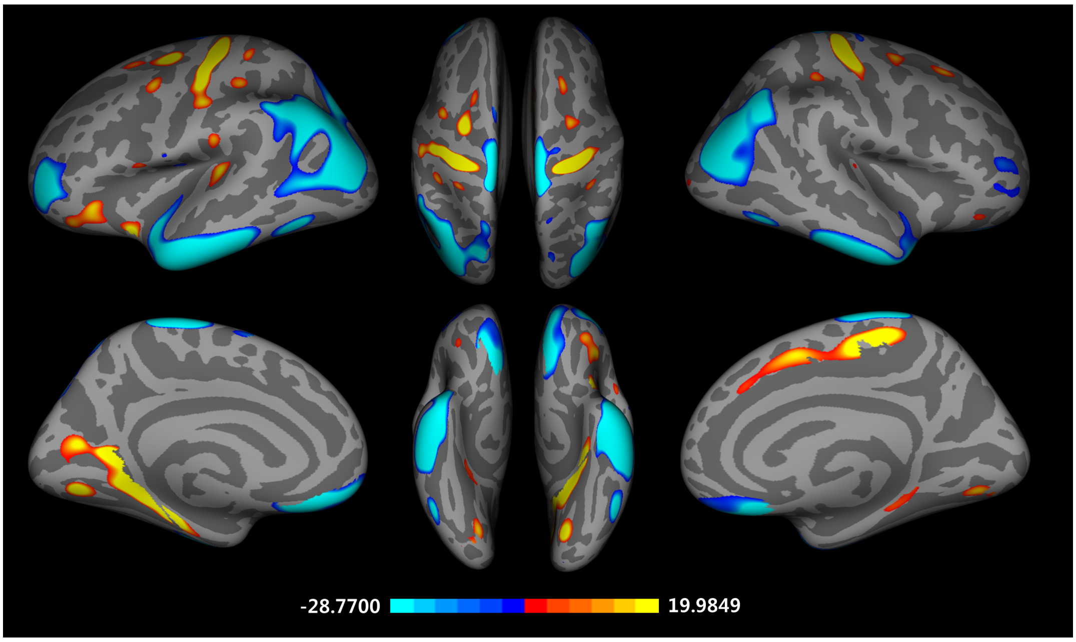

(B) Images smoothed at 20mm full width at half maximum

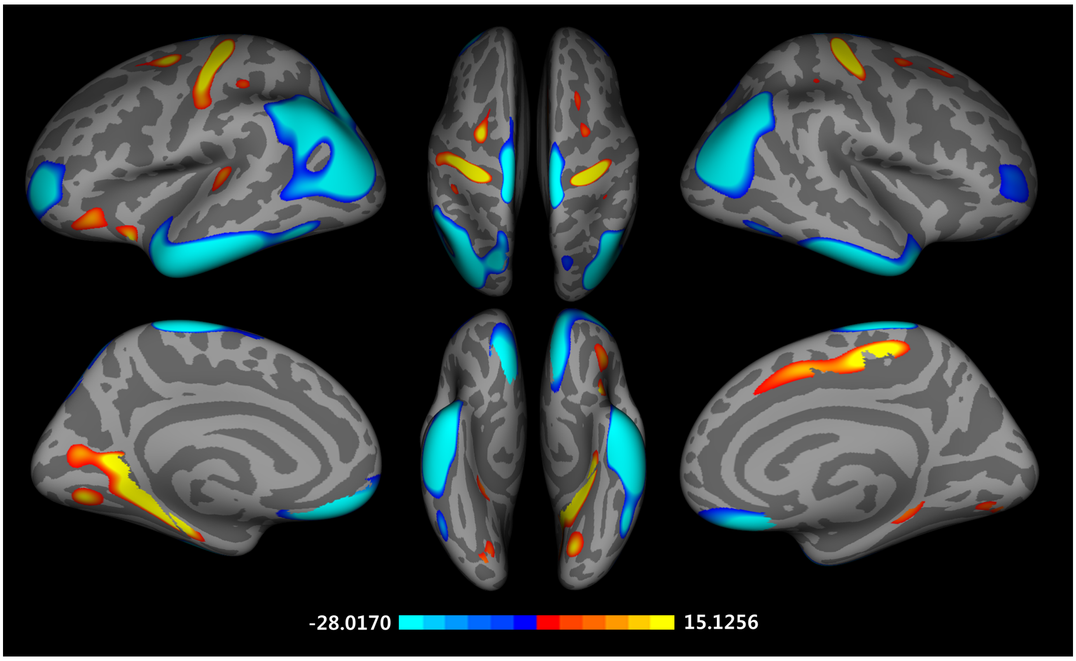

These images were created using FreeSurfer software (Version 6.0.0, http://surfer.nmr.mgh.harvard.edu/)

Supplementary Figure S3 Vertex-wise group differences in cortical surface area adjusted for the effects of age, education years, MMSE scores, MR vendors, and total surface area (T-map, thresholded at FDR corrected $p < 0.05$, Caucasian-East Asian)

(A) Images smoothed at 15mm full width at half maximum

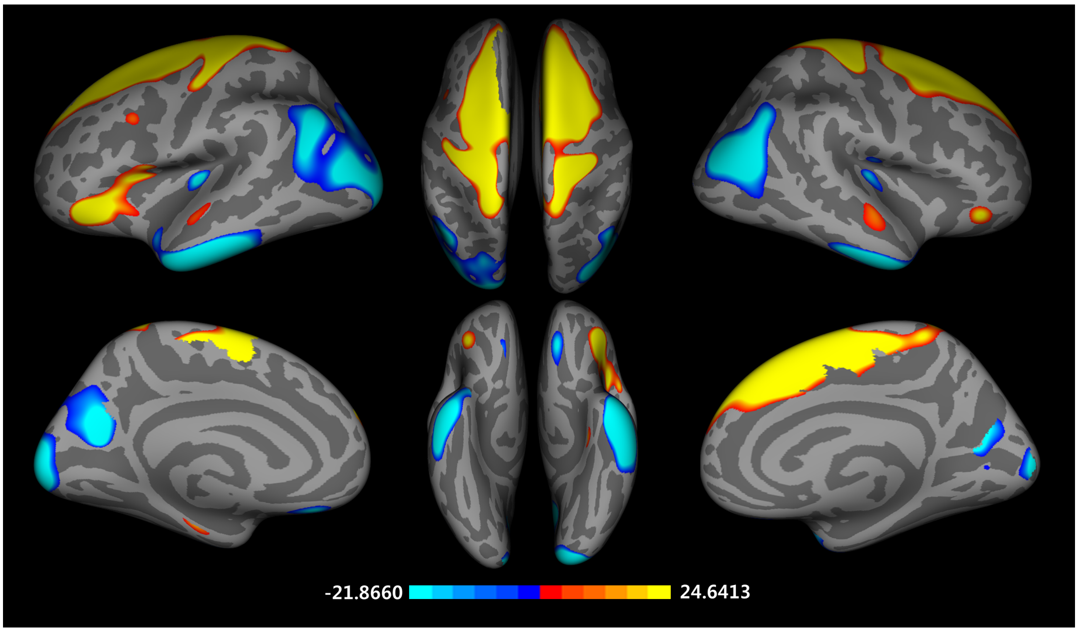

(B) Images smoothed at 20mm full width at half maximum

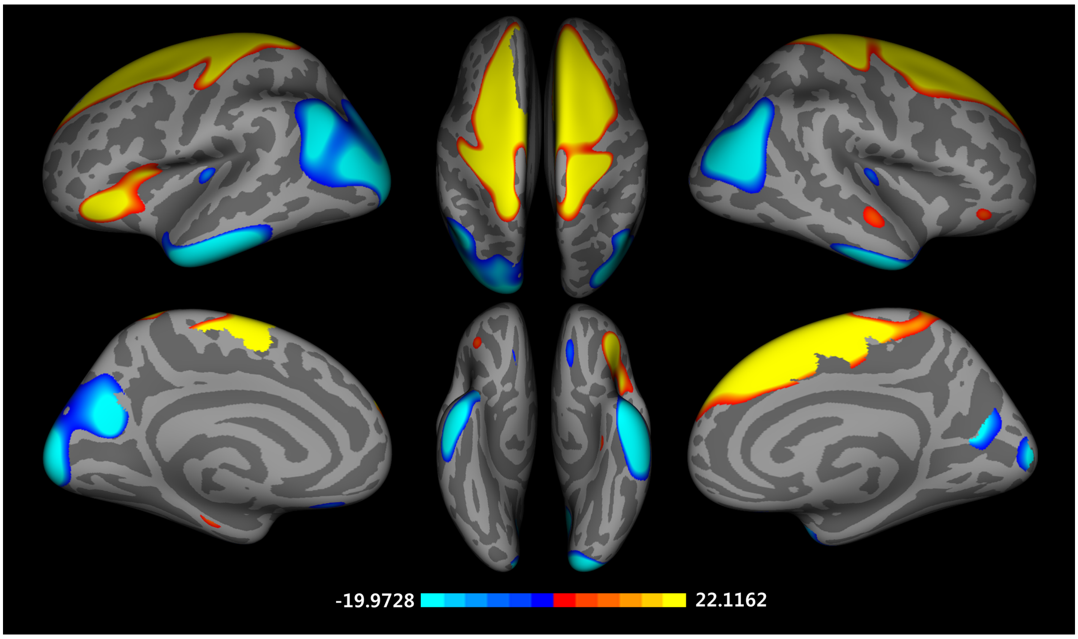

These images were created using FreeSurfer software (Version 6.0.0, http://surfer.nmr.mgh.harvard.edu/)
